# The Importance of Gender-Neutral Terminology in Risk Evaluation and Mitigation Strategy Programs: A Call to Action

**DOI:** 10.2196/45329

**Published:** 2023-06-22

**Authors:** Colin Burnette, William Smithy, Daniel Strock, Torunn E Sivesind, Robert Dellavalle

**Affiliations:** 1 Nova Southeastern University College of Osteopathic Medicine Davie, FL United States; 2 Eastern Virginia Medical School Norfolk, VA United States; 3 University of Colorado School of Medicine Aurora, CO United States; 4 US Department of Veterans Affairs University of Colorado Aurora, CO United States

**Keywords:** iPLEDGE, REMS, evaluation and mitigation strategy, gender dysmorphia, transgender patients, call to action, oral retinoid, medical community, gender, identity, biological sex, accessibility, barrier, gender diversity, quality of care, treatment

## Abstract

The use of risk evaluation and mitigation strategy (REMS) programs is frequently required for prescriptions with potentially teratogenic effects, especially in the field of dermatology. Among these REMS programs, the most well-known example is isotretinoin, an oral retinoid that uses the iPLEDGE system. iPLEDGE has strict regulations and a lengthy approval process, and until recently, patients were grouped into 3 categories: male, female, or female of reproductive potential. This strict grouping has posed problems in the medical community, especially for gender-diverse individuals where their perceived gender conflates with their assigned grouping causing patient-specific distress. The distinction between gender—a multifactorial perception of identity—and biological sex is addressed under new iPLEDGE guidelines. Dermatologists now register patients under one of 2 categories: patients who can become pregnant and those who cannot become pregnant. This change simultaneously improves the accessibility to isotretinoin among gender-diverse individuals, while limiting prescription barriers. Despite initial success being limited due to lengthy system conversions, a registration process based on reproductive potential ultimately enhances iPLEDGE’s goal to prevent potential birth defects. We propose that other REMS programs follow the standard set by the iPLEDGE system, including those for the medications thalidomide, acitretin, and mycophenolate mofetil, all of which currently have a similar taxonomy to that of the old iPLEDGE system. Implementing the standardization of gender-neutral terminology can maximize enrollment and minimize distress. Current and ongoing refinement of iPLEDGE and other REMS is needed to build protocols solely around the prevention of birth defects without regard to sex or gender.

## Introduction

Risk evaluation and mitigation strategy (REMS) programs in dermatology should clarify how they define gender and biological sex to appropriately focus on patients’ reproductive potential and risk for birth defects (Table 1). Specifically, REMS programs should adopt proper gender-neutral terminology. While several teratogenic prescriptions are monitored by REMS programs, isotretinoin, an oral retinoid used for acne treatment is likely the most well-known. Regulated by iPLEDGE, isotretinoin prescription originally required patients to register under one of 3 categories: male, female, or female of reproductive potential [1]. This classification system posed problems as providers were required to categorize patients of gender-diverse backgrounds into limited categories. For example, transgender males with intact uteruses and ovaries might have been categorized as females of reproductive potential. In some cases, patients have forgone treatment because improper categorization conflicted with their gender identity [2].

**Figure 1:**
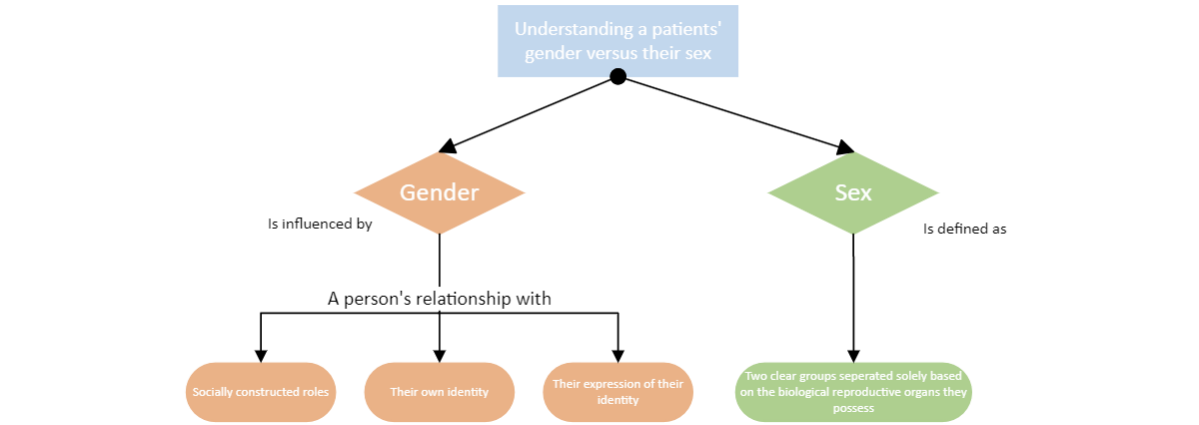
Flowchart breaking down the differences between gender and identity.

Medical care providers may conflate gender and biological sex. Growing awareness and inadequate terminology for transgender patients have led to changes. In December 2021, the Food and Drug Administration streamlined the iPLEDGE process and introduced gender-neutral terminology, allowing for the separation of a patient’s biological sex from their gender identity (Figure 1). Dermatologists now register patients under one of 2 categories: patients who can become pregnant and those who cannot become pregnant [3,4]. This change may improve access to isotretinoin for transgender candidates such as transmasculine patients with an increased predisposition for acne secondary to exogenous testosterone therapy [5]. A registration process based on reproductive potential ultimately enhances iPLEDGE’s goal to prevent potential birth defects.

**Table 1:** Definitions of key terms.

Keyword	Definition
Gender	Socially constructed characteristics of women, men, girls, and boys, which define their identity
Sex	A set of biological attributes in humans and animals
Gender incongruence	Gender identity that is different from a person’s biological sex
Gender-diverse	Gender identity that demonstrates a diversity of expression beyond the binary framework
Transgender	Denoting or relating to a person whose gender identity does not correspond with the sex registered to them at birth
Transmasculine	A person who was registered as female at birth but whose gender identity is characterized or aligned with masculinity
Transfeminine	A person who was registered as male at birth but whose gender identity is characterized or aligned with femininity
Female of reproductive potential	A person who is capable of giving birth to offspring

## Conclusions

Though recent changes to iPLEDGE do not resolve population-specific concerns faced by gender-diverse individuals, it is nonetheless a pivotal step in transgender patient care—an additional step that other REMS programs have yet to adopt. Prescription medications including thalidomide, acitretin, and mycophenolate mofetil still use a similar taxonomy to the old iPLEDGE system, with patient groupings of male, female, or female of reproductive potential [6,7]. Nevertheless, overall prescription prevalence and increasing associations of skin disease with exogenous hormone therapy indicate a potential area for the use of updated REMS terminology [8]. Future standardization of gender-neutral terminology can maximize patient enrollment, minimize distress, and prevent teratogen exposure. The revision of iPLEDGE categories with gender-neutral terminology reveals continued inadequacies; ongoing effort is needed to build REMS protocols solely around the prevention of birth defects, without regard to sex or gender.

## Abbreviations

REMS: risk evaluation and mitigation strategy
